# The fifth leaf and spike organs of barley (*Hordeum vulgare* L.) display different physiological and metabolic responses to drought stress

**DOI:** 10.1186/s12870-016-0922-1

**Published:** 2016-11-09

**Authors:** Jordan A. Hein, Mark E. Sherrard, Kirk P. Manfredi, Tilahun Abebe

**Affiliations:** 1 Department of Biology, University of Northern Iowa, Cedar Falls, IA 50614 USA; 2 Department of Chemistry and Biochemistry, University of Northern Iowa, Cedar Falls, IA 50614 USA

**Keywords:** Barley, Awn, Leaf, Lemma, Palea, Drought, Water status, Gas exchange, Metabolites

## Abstract

**Background:**

Photosynthetic organs of the cereal spike (ear) provide assimilate for grain filling, but their response to drought is poorly understood. In this study, we characterized the drought response of individual organs of the barley spike (awn, lemma, and palea) and compared them with a vegetative organ (fifth leaf). Understanding differences in physiological and metabolic responses between the leaf and spike organs during drought can help us develop high yielding cultivars for environments where terminal drought is prevalent.

**Results:**

We exposed barley plants to drought by withholding water for 4 days at the grain filling stage and compared changes in: (1) relative water content (RWC), (2) osmotic potential (Ψ_s_), (3) osmotic adjustment (OA), (4) gas exchange, and (5) metabolite content between organs. Drought reduced RWC and Ψ_s_ in all four organs, but the decrease in RWC was greater and there was a smaller change in Ψ_s_ in the fifth leaf than the spike organs. We detected evidence of OA in the awn, lemma, and palea, but not in the fifth leaf. Rates of gas exchange declined more rapidly in the fifth leaf than awn during drought. We identified 18 metabolites but, only ten metabolites accumulated significantly during drought in one or more organs. Among these, proline accumulated in all organs during drought while accumulation of the other metabolites varied between organs. This may suggest that each organ in the same plant uses a different set of osmolytes for drought resistance.

**Conclusions:**

Our results suggest that photosynthetic organs of the barley spike maintain higher water content, greater osmotic adjustment, and higher rates of gas exchange than the leaf during drought.

**Electronic supplementary material:**

The online version of this article (doi:10.1186/s12870-016-0922-1) contains supplementary material, which is available to authorized users.

## Background

Drought reduces crop yield more than any other environmental factor [1, 2]. Plants are particularly sensitive to drought during the reproductive stage of their life cycle [3–5]. Pre-anthesis drought can cause sterility and senescence of flowers [3] and post-anthesis drought can reduce seed size [6, 7]. The effect of drought on cereal crops has been well-studied but most research has focused on vegetative structures (i.e., leaves). Comparatively little is known about the response of the photosynthetic organs in the spike (ear) to drought. The spike is an important supplier of assimilate for seed development [8–10].

Barley (*Hordeum vulgare* L.) is an important malting, food, and feed crop [11] and ranks fourth in global production among cereal crops behind corn, paddy rice, and wheat [12]. Because barley originated in a semi-arid region, known historically as the Fertile Crescent [13], it is relatively resistant to periods of water shortage [14]. Barley displays three strategies for coping with drought [15, 16]: escape, avoidance, and tolerance. Varieties from regions characterized by terminal drought (drought at the reproductive stage) complete their life cycle before the onset of severe water deficit [17–20], which is consistent with a drought escape strategy [21, 22]. By contrast, plants using a drought avoidance strategy maintain sufficient cellular hydration when water is scarce [21–23]. Common drought avoidance mechanisms in barley include minimizing water loss via stomatal control [24], production of extensive root system to extract soil moisture [25, 26], and altering metabolism to accumulate compatible solutes (osmolytes) for osmotic adjustment [27, 28]. Drought tolerant varieties maintain physiological functions at low tissue water potentials [21, 22]. Typical drought tolerance mechanisms in barley include synthesis of proteins and compatible solutes to detoxify reactive oxygen species (ROS) and stabilize macromolecules and membranes [29–32] and mobilization of stem reserves (e.g., glucose, fructose, sucrose, and fructans) to supply carbon for grain filling [33–36]. These three contrasting strategies can also be used in combination [15], highlighting the complexity of drought response in barley and the challenges associated with developing cultivars for dry environments.

Drought resistance in barley is controlled by several genes. Transcriptome studies have shown that genes for heat shock proteins (chaperones), late-embryogenesis abundant (LEA) proteins, osmolyte biosynthesis, ROS scavenging, signal transduction, defense, and others are up-regulated in response to drought [37–41]. These changes at the transcription level also increase accumulation of proteins and metabolites involved in drought resistance [42–45].

The spike organs of barley (lemma, palea, and awn) are photosynthetically active and contribute as much as 76 % of the dry weight of the kernel [46–48]. Because of its larger size, the awn can account for up to 90 % of spike photosynthesis in barley under normal conditions [49]. The spike is resistant to drought and spike photosynthesis is particularly important for grain filling during shortages of water. The spike has several attributes that confer resistance to drought stress. Relative to the leaf, the spike has better CO_2_ diffusive conductance during drought [9], suggesting efficient assimilation of CO_2_ per unit of water transpired [9, 50, 51]. The spike has better osmotic adjustment [52], delayed senescence [53, 54], a greater capacity to transport assimilate [54], and a photosynthetic metabolism suspected to be intermediate between C_3_ and C_4_ pathways [54]. Further, the lemma and palea tightly enclose the developing kernel and recycle respired CO_2_ [9, 51, 53, 55]. The significance of the spike for grain filling is amplified during drought [9, 10, 56] with some authors suggesting that spike photosynthesis can be used as a selection tool for developing drought resistant cereals [53, 57, 58].

Emerging evidence also suggests that the various organs of the barley spike respond differently to drought. Transcriptome analysis by our group found that drought alters expression of more genes in the awn than the lemma, palea, and kernel [59]. However, it is not clear whether these changes at the transcription level lead to accumulation of proteins and metabolites required for drought resistance. In this study, we examined whether metabolite accumulation in response to drought at the early stages of grain filling differs between the fifth (penultimate) leaf and spike organs (lemma, palea, awn) of barley using non-targeted metabolite profiling. We also compared the water status and gas exchange of these photosynthetic organs during drought. To our knowledge, this is the first study to compare physiological and metabolic changes in individual spike organs and leaf of barley in response to terminal drought. Understanding differences in physiological and metabolic responses between the leaf and spike organs during drought can help us develop better approaches to increase yield of cereals in environments where terminal drought is prevalent.

## Methods

### Plant materials and growth conditions

We used a six-row, drought tolerant [60] barley variety (*Hordeum vulgare* L. var. Giza 132) for this study. The seeds were obtained from the National Small Grains Collection of the United States Department of Agriculture, Aberdeen, Idaho. We grew plants in 2.5 L pots (16 cm top diameter × 12 cm bottom diameter × 17 cm height) filled with 800 g of soil (17 % topsoil, 50 % Canadian peat moss, 25 % vermiculite, and 8 % rice hulls). Before planting the seeds, the soil was saturated with water to a total weight of 1200 g. In each pot, we planted eight seeds, two cm deep, with the awn end up in an evenly-spaced, circular pattern. Then, 5 g of Osmocote® (Scotts Company LLC, Marysville, OH) slow release fertilizer (N-P-K 19-6-12) was added. All planting occurred between 0900 and 1000 CST (3–4 h into the photoperiod).

We grew the plants in a controlled growth chamber (Conviron CMP-6050 connected to a Thermoflex 10,000 chiller) under conditions of 16 h photoperiod, 22 °C days/18 °C nights, and 60 % relative humidity. In the morning, we stepped up light intensity (219, 437, 656, and 715 μmoles m^−2^ sec^−1^) in half hour intervals and at the end of the day, we stepped down light intensity in the same manner. We fertilized each pot with 100 mL of 4 g/L Jack’s Professional with magnesium (N-P-K 20-19-20) twice: (1) one week after planting and (2) two weeks before samples were collected. At Zadoks stage 12 (second leaf unfurled) [61], we thinned the number of seedlings to five per pot to ensure a uniform stand. For the first 3 weeks after planting, we watered all pots to a final weight of 1200 g every other day to promote seedling establishment. After 3 weeks, we watered all pots to a final weight of 1200 g daily until commencing the drought treatment. All watering occurred between 0900 and 1000 CST (3–4 h into the photoperiod.

### Drought treatment

At Zadoks stage 71 (kernel watery ripe) [61], plants were randomly assigned to either the “control” group or the “stressed” group. Control pots were watered to 1200 g total weight each day. Plants in the stressed group were exposed to drought by withholding water for 4 days. More specifically, stressed pots were weighed each day and water was added to bring the weight of each pot to that of the heaviest stressed pot, which was 900 g (day 1), 790 g (day 2), 630 g (day 3), and 580 g (day 4).

### Experimental design

We examined changes in water status (relative water content, osmotic potential, osmotic adjustment), gas exchange (photosynthesis and stomatal conductance), and metabolite content in the fifth (penultimate) leaf and spike organs of barley during drought. Measurements of relative water content (RWC), osmotic potential (Ψ_s_), and gas exchange are based on three replicates (pots) using a completely randomized design. Specifically, we randomly selected one plant from three different pots for each treatment, measured gas exchange on the fifth leaf and awns, and then harvested the fifth leaf and spike organs (awn, lemma, palea) of that plant to quantify RWC. We repeated this protocol every day of the 4-day drought treatment using the remaining plants in each pot.

Measurements of osmotic potential and metabolite accumulation are based on six replicates (blocks) using a randomized complete block design. The six replicates were planted on different days due to space limitation. We harvested the fifth leaf and spike organs (awn, lemma, palea) on the fourth day of drought stress for analysis. The main experimental factors used for analysis were treatment (control vs. stressed) and organ type. Date of planting was included as a random (block) factor.

### Relative water content

We measured relative water content (RWC) of the fifth (penultimate) leaf, awn, lemma, and palea of control and stressed plants each day of the 4-day drought treatment. Each day, we harvested the four organs and immediately recorded their fresh weight. Next, we submerged each organ in 15 mL of distilled water in a 100 × 15 mm Petri dish and placed them in darkness for 24 h at 4 °C. We want to point out that the tips of the leaves and the awns become progressively discolored as drought gets more severe. As a result, RWC was measured from the basal, green portion of the fifth leaf and awn. By the end of the 4-day treatment, about a quarter of the tip of the leaf and awn was discolored in the stressed plants and were not included in all measurements. The fifth leaf and awns were cut into ~ one cm segments to facilitate diffusion of water. The next day, we measured turgid weight after removing all traces of water on the surface of the samples using a Buchner funnel and gentle vacuum. Each organ was then dried at 70 °C for 24 h and dry weights were measured. We calculated RWC from fresh, turgid, and dry weights using the equation:

$$ RWC = \frac{\left( Fresh\  weight - Dry\  weight\right)}{\left( Turgid\  weight - Dry\  weight\right)} \times 100 $$

### Osmotic potential

We measured osmotic potential (Ψ_s_) of the fifth leaf, awn, lemma, and palea of control and stressed plants on the fourth day of drought treatment. Organs were harvested, frozen in liquid nitrogen, and stored at −80 °C prior to analysis. Each frozen sample was transferred to a 0.5 mL centrifuge tube with a hole in the bottom. The tube was placed into another 1.5 mL tube and centrifuged at 12,000 × g for 10 min. We used 10 μL of the sap to measure osmolality using a vapor pressure osmometer (Vapro® 5520, Wescor, Inc. Logan, Utah). Osmolality values were converted to osmotic potential using the formula:

$$ {\varPsi}_s=-c\times 2.5 \times {10}^{-3} $$

where *Ψ*_*s*_ is osmotic potential in megapascals (MPa) and *c* is osmolality of the sap in mosmol kg^−1^ [62].

### Osmotic adjustment

Osmotic adjustment (OA) is the lowering of Ψ_s_ due to net solute accumulation in response to water deficit. We measured OA of the fifth leaf, awn, lemma, and palea on the fourth day of drought stress according to the rehydration method [63–65]. In brief, we calculated OA for each organ as the difference between Ψ_s_ of the control tissue at full turgor and Ψ_s_ of stressed tissue at full turgor. Ψ_s_ at full turgor was measured after rehydrating control and stressed samples in 15 mL of distilled water in a 100 × 15 mm Petri dish for 24 h in darkness at 4 °C. All traces of surface water were removed from the samples using a Buchner funnel and gentle vacuum. The samples were frozen in liquid nitrogen and stored at −80 °C until needed. We then thawed the samples, extracted the sap, and measured osmolality (see osmotic potential measurement above for methods) with a vapor pressure osmometer (Vapro® 5520, Wescor Inc., Logan, Utah).

### Gas exchange

Each day of the drought treatment, we randomly selected one plant from three control and three stress treatment pots and measured photosynthesis (*A)* and stomatal conductance (*g*_S_) of the fifth leaf and the awns using an open gas-exchange system (LI-6400, Li-COR Inc., Lincoln, NE). For the fifth leaf, we measured gas exchange at a controlled cuvette temperature of 22 °C, a vapor pressure deficit of 1.5 – 1.7 kPa, and a saturating irradiance of 2000 μmol m^−2^ s^−1^. For the awns, we measured gas exchange using the needle gasket of the LI-6400. Measurements were made on two awns of the fourth spikelet (from the base of the inflorescence) under the same cuvette conditions as the fifth leaf except the vapor pressure deficit was set to ~2.5 kPa. All measurements were made between 0900 and 1000 CST (3–4 h into the photoperiod). After recording the gas exchange measurements, leaves and awns were harvested to determine surface area. We measured leaf area using a digital caliper. The 3 cm region of the awn we used for gas exchange resembles a triangular prism with a 120° angle on the abaxial surface and 30° angles on each corner of the adaxial surface [66]. Therefore, awn area was calculated by measuring the width of the adaxial surface in imageJ (http://imagej.nih.gov/ij/) and calculating the width of the remaining sides using these angles.

### Metabolite extraction, derivatization, and analysis

To analyze metabolites, we harvested the fifth leaf, awn, lemma, and palea from three-four plants per pot on the fourth day of drought treatment between 1100 and 1300 CST (5–7 h into the photoperiod). The three lowest and three highest spikelets on the spike were excluded from this analysis. We also removed 1 cm from the base and 2 cm from the tip of the awn (because of discoloration in the tip of stressed plants) and 2–3 cm from the tip of the leaf (because of senescence in stressed plants). The samples were frozen in liquid nitrogen and stored at −80 °C.

We ground the frozen samples in liquid nitrogen using a mortar and pestle and a 100 mg sub-sample was used for extraction and derivatization of polar metabolites according to Lisec et al., [67]. A solution of ribitol (60 μl of 20 μg/ml stock) was added as internal standard. The derivatized extract was dried under vacuum, dissolved in 200 μl chloroform, and transferred to a 300 μL GC vial. One μL of sample was injected into an Agilent 6890 GC instrument (Agilent, Santa Clara, CA) equipped with a Hewlett Packard 5973 MSD and a Restek Rtx®-5MS–Low-Bleed GC-MS Column. The instrument was set at 230 °C, in split mode, with a split ratio of 16.5:1. The oven was set to an initial temperature of 80 °C. After holding for 2 min, the temperature was increased at a rate of 9 °C per min to a final temperature of 290 °C. The system was held at 290 °C for 6 min. Helium was used as the carrier gas and set to a flow rate of 1.2 mL/min. Gaseous compounds eluted from the GC were fed into an Agilent 5973 mass spectrometer (Agilent, Santa Clara, CA) and bombarded by an electron impact (EI) ionization source with an ionization energy of −70 eV at a temperature of 200–250 °C for further separation based on mass-to-charge ratio. Ions were detected on a quadrupole mass selective detector.

Acquired spectra were deconvoluted, quantified, and identified using AMDIS (Automated Mass Spectral Deconvolution and Identification System, http://chemdata.nist.gov/dokuwiki/doku.php?id=chemdata:amdis). Initially, we matched peaks to spectra from the National Institute of Standards and Technology (NIST) MS Search 2.0 mass spectral database. We used authentic targets and standard libraries to confirm peak identities in AMDIS. In addition to the RI (relative intensity) function in AMDIS, we converted the output from AMDIS to a spreadsheet and verified the RI manually. The integrated signal (after deconvolution) for ribitol was divided by the integrated signal for each metabolite within the injection to get relative amounts (response ratio).

### Statistical analysis

We analyzed RWC, photosynthesis, and stomatal conductance data using repeated measures ANOVA with three factors: treatment (control vs. stress), organ type, and time (day of treatment). Treatment and organ were between-subject factors and time was the repeated measures factor (within-subject factor). Variation between pots (nested within treatment) was included as a random factor. This analysis is represented by the linear model:

$$ {y}_{ijkl} = \mu + treatmen{t}_i + orga{n}_j + treatment\times orga{n}_{ij} + tim{e}_k + treatment\times tim{e}_{ik} + orga n\times tim{e}_{jk} + treatment\times orga n\times tim{e}_{ijk} + po{t}_{l(i)}+{\varepsilon}_{ijkl} $$

where *y*_*ijkl*_ is the response at treatment level *i*, in organ *j*, at time *k*, and in pot *l; μ* is the mean of each treatment combination, pot_*l(i)*_ is experimental error due to the effect of pot *l* receiving treatment *i*, and *ε*_*ijk*_ is sampling error due to variation among plants within pots. The model assumes there is no time × pot interaction. Treatment, organ type, time, and their interactions are fixed effects and pot_*l(i)*_ and *ε*_*ijk*_ are random effects. ANOVAs with repeated measures are particularly susceptible to violating the assumption of sphericity, the condition where differences between pairs of repeated measures factors have equal variance and equal covariance. We tested four covariance structures to assess correlations between levels of the repeated measures factor (time): compound symmetric (CS), autoregressive order one (AR(1)), Huynh-Feldt (HF), and unstructured (UN). AR(1), HF, and UN failed to converge so significance tests were performed based on CS. For interaction effects, we used Tukey’s pairwise comparison to determine differences between pairs of treatment × organ combinations at each time point.

To determine differences in Ψ_s_ and metabolite accumulation in response to drought, we used the linear model:

$$ {y}_{ijk} = \mu + treatmen{t}_{i(k)} + orga{n}_j + bloc{k}_k + treatmen t\times orga{n}_{ij}+{\varepsilon}_{ij} $$

where *y*_*ijk*_ is the response at treatment level *i* , organ *j*, and block *k*, *μ* is the overall mean and *ε*_*ij*_ is the deviation for *ij* ^th^ subject. In this model there is no treatment × block interaction and variance from block to block is assumed to be constant. We then used Tukey’s pairwise comparison to further examine the treatment effect in each organ.

We tested the assumptions of normality and homoscedasticity (equal variance) in ANOVA using PROC UNIVARIATE and Levene’s test with option TYPE BF in PROC GLM. These tests revealed that RWC and gas exchange data were normally distributed with homogeneous variance. Accordingly, we performed the repeated measures ANOVA on untransformed data using the REPEATED statement in PROC MIXED. Osmotic potential and the metabolite data were neither normally distributed nor of constant variance. We corrected non-normality and heterogeneous variance using the Box-Cox power transformation. This transformation improved variability in the data but a few metabolites were still heterogeneous. Because ANOVA is robust to non-normal and heteroscedastic data, we tested mean differences in PROC MIXED using the transformed osmotic potential and metabolite data. All statistical analyses were performed in SAS v. 9.4 (SAS Institute Inc., Cary, NC).

## Results

### Water status of the fifth leaf and spike organs during drought

**Fig. 1:**
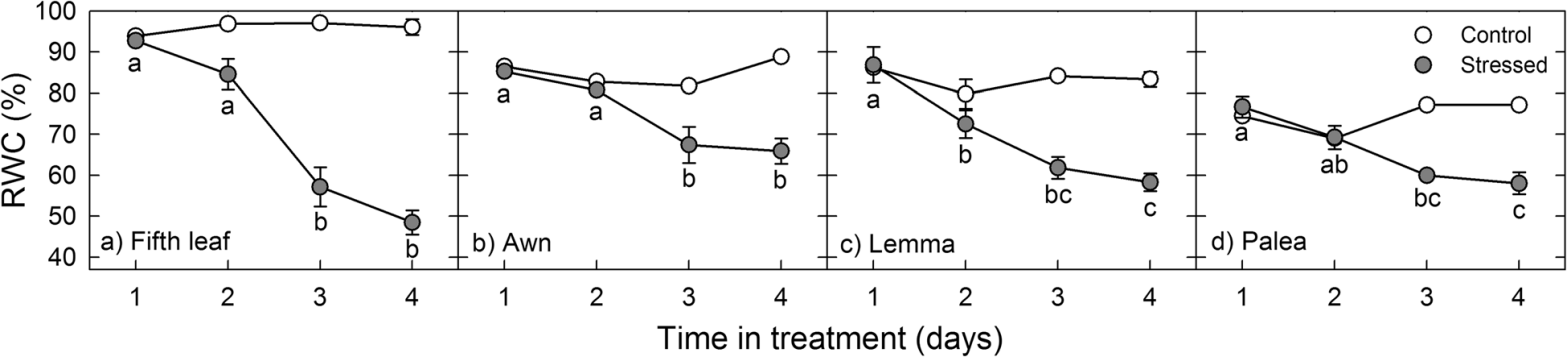
Effect of drought on relative water content (RWC) in the fifth leaf and spike organs. RWC was measured in the fifth leaf (**a**), awn (**b**), lemma (**c**), and palea (**d**) over the 4-day drought treatment during grain filling. Significant differences between days are indicated with *lower case letters* (stressed plants). For control plants, RWC did not differ significantly between days in any organ. Data are presented as the mean of three replicates ± SE. We used SigmaPlot 10.0 (Systat Software Inc., San Jose, CA) to make the figures

Relative water content (RWC) differed significantly between treatments (control vs. stressed plants), organs, time (day of treatment), and their interactions (Additional file 1: Table S1). In control plants, RWC did not vary between days in any organ during the treatment period (Fig. 1). Average RWC was highest in the fifth leaf (96 %), followed by the awn (85 %), lemma (83 %), and palea (74 %). In stressed plants, RWC declined progressively during the treatment period in every organ (Fig. 1). In stressed fifth leaves, RWC decreased from 94 to 49 % (Fig. 1a), which was the largest loss of water in any organ. By the fourth day of treatment, the leaves of stressed plants were severely wilted. In stressed awns, RWC decreased from 85 to 66 % (Fig. 1b), which was the smallest loss in RWC of any organ. In stressed lemmas, RWC decreased from 83 to 58 % (Fig. 1c) and in stressed paleas, RWC decreased from 77 to 58 % (Fig. 1d).

**Fig. 2:**
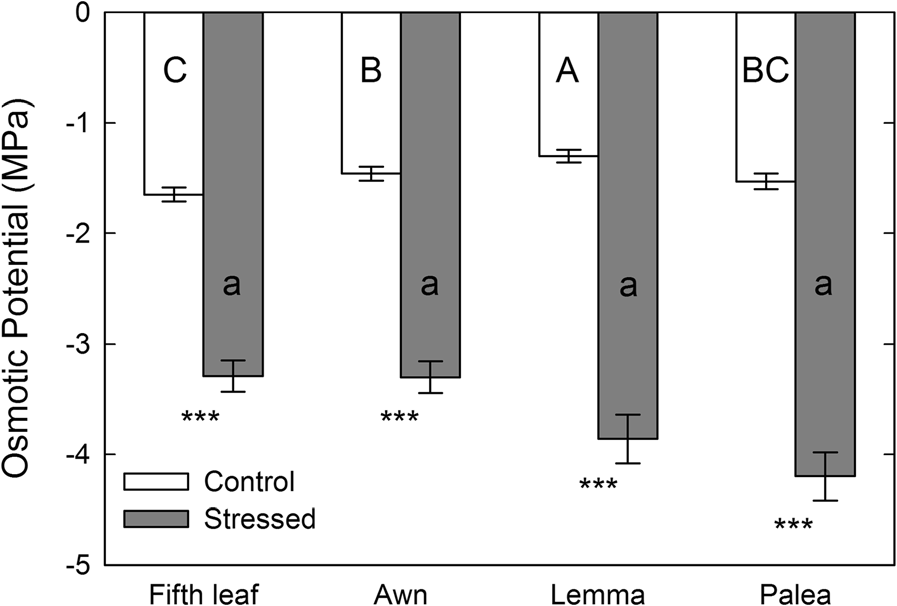
Changes in osmotic potential in the fifth leaf and spike organs of barley during drought. Osmotic potential was measured on the fourth day of drought treatment during grain filling. Significant differences between organs are indicated with *lower case letters* (stressed plants) and *upper-case letters* (control plants). Within a given organ, significant differences between treatments (control vs. drought) are indicated with asterisks, where * = *P* < 0.05, ** = *P* < 0.01, and *** = *P* < 0.001. Data are presented as the mean of six replicates ± SE. We used SigmaPlot 10.0 to make the figure

Osmotic potential (Ψ_s_) differed significantly between treatments, organs, and their interaction (Additional file 1: Table S1). In control plants, Ψ_s_ was lowest in the fifth leaf (−1.65 MPa) followed by the palea (−1.53 MPa), awn (−1.46 MPa), and lemma (−1.3 MPa, Fig. 2). Drought significantly reduced Ψ_s_ in every organ (Fig. 2). After the 4-day drought treatment, Ψ_s_ had dropped to −3.3 MPa in the fifth leaf and awn, −3.86 MPa in the lemma, and −4.2 MPa in the palea. All three spike organs, showed evidence of osmotic adjustment (range = 0.30 – 0.36 MPa; Table 1), which is an indicator of ability to maintain cellular water during drought. There was no evidence of osmotic adjustment in the fifth leaf (Table 1) and on the fourth day of drought it showed severe wilting.

**Table 1:** Osmotic adjustment (OA) in the spike organs and the fifth leaf of barley on the fourth day of drought treatment

Organ	OA
Fifth leaf	−0.16 ± 0.07
Awn	0.36 ± 0.07
Lemma	0.42 ± 0.05
Palea	0.30 ± 0.04

Values are the mean of three replicates ± SE

### Gas exchange in the fifth leaf and awn during drought

**Fig. 3:**
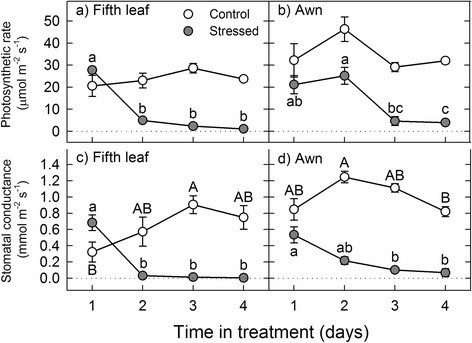
Effect of drought on gas exchange in the fifth leaf and awn of barley. Photosynthesis and stomatal conductance in the fifth leaf (**a** and **c**) and awn (**b** and **d**) were measured over the four-day drought treatment at the grain filling stage. Significant differences between days are indicated with *lower case letters* (stressed plants) and *upper-case letters* (control plants). Data are presented as the mean of three replicates ± SE. We used SigmaPlot 10.0 (Systat Software Inc., San Jose, CA) to make the figures

Photosynthetic rate (*A*) and stomatal conductance (*g*_s_) differed significantly between treatments, organs, and times (Additional file 1: Table S1). We detected significant treatment × organ, time × organ, and time × treatment terms for *g*_s_ and significant time × organ, and time × treatment terms for *A* (Additional file 1: Table S1). We also detected a significant time × treatment × organ interaction for *g*_s_ but not *A* (Additional file 1: Table S1). In both the awn and fifth leaf, *A* and *g*_s_ remained stable in control plants throughout the treatment period (Fig. 3). In stressed fifth leaves, *A* and *g*_s_ declined significantly on the second day of drought treatment and remained low there after (Fig. 3a, c). In stressed awns, by contrast, *A* and *g*_s_ did not decline significantly until the third day of the drought treatment (Fig. 3b, d).

### Metabolic changes in the fifth leaf and spike organs during drought

**Fig. 4:**
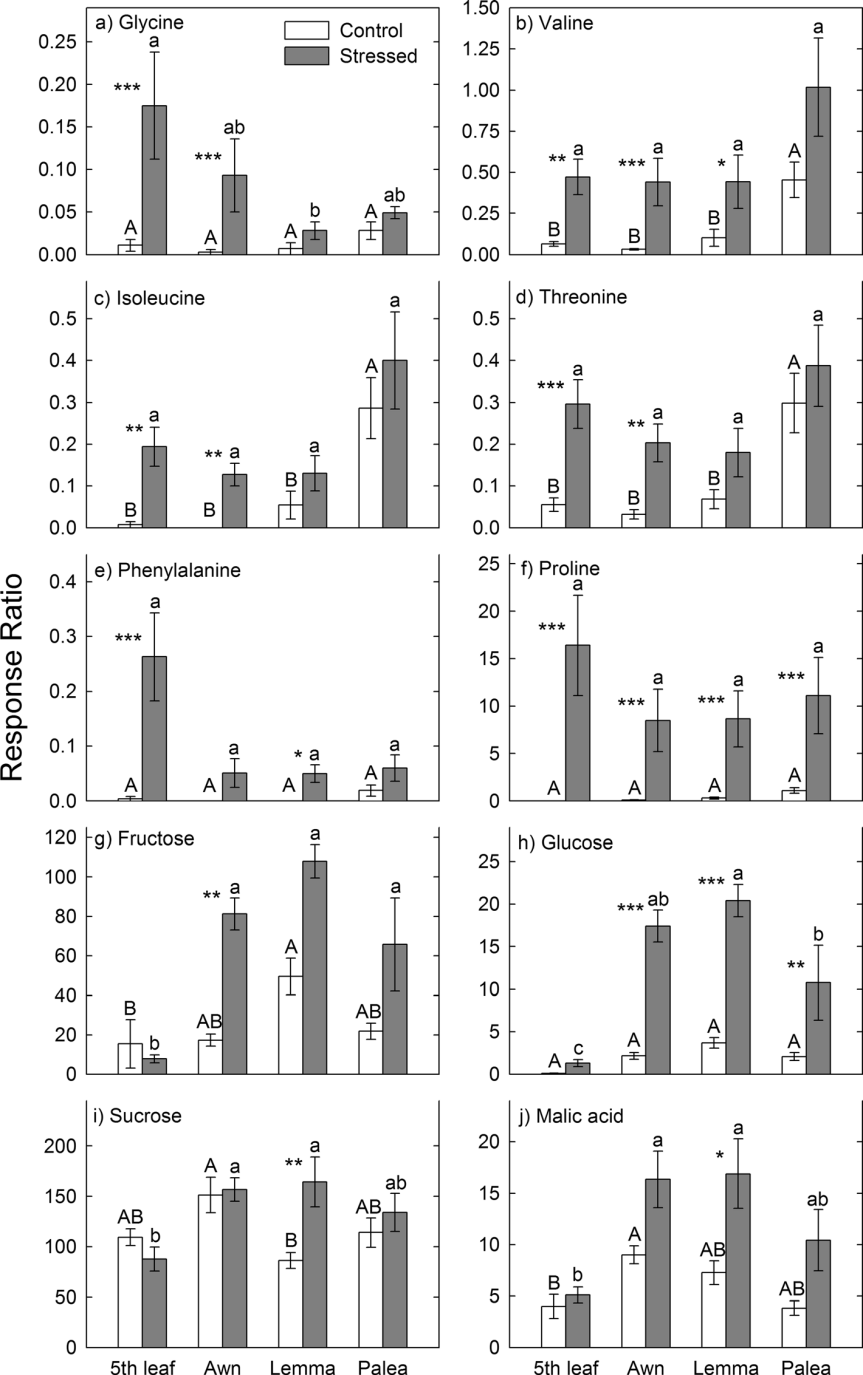
Metabolic changes in the fifth leaf and spike organs of barley during drought. Metabolites were measured on the fourth day of drought treatment during grain filling. Significant differences between organs are indicated with *lower case letters* (stressed plants) and *upper-case letters* (control plants). Within an organ, significant differences between treatments (control vs. drought) are indicated with asterisks, where * = *P* < 0.05, ** = *P* < 0.01, and *** = *P* < 0.001. Data are presented as the mean of six replicates ± SE. We used SigmaPlot 10.0 (Systat Software Inc., San Jose, CA) to make the figures

We identified 18 metabolites but only ten metabolites accumulated significantly during drought in one or more organs (Fig. 4, Additional file 1: Table S2): six amino acids (Fig. 4a–f), three sugars (Fig. 4g–i), and one organic acid (Fig. 4j). Although there was no evidence of osmotic adjustment in the fifth leaf, it accumulated six metabolites during drought. The awn, lemma, and palea accumulated seven, six, and two metabolites during drought, respectively (Fig. 4).

Metabolites representing five different families of amino acids accumulated in the photosynthetic organs during drought: serine (glycine), branched-chain (valine and isoleucine), aspartate (threonine), glutamine (proline), and aromatic amino acids (phenylalanine; Fig. 4). Proline was the only amino acid that accumulated in all organs during drought (Fig. 4f). Valine accumulated in the fifth leaf, awn, and lemma during drought (Fig. 4b). Glycine, isoleucine and threonine accumulated in the fifth leaf and awn during drought (Fig. 4a, c, d). Phenylalanine accumulated in the fifth leaf and lemma during drought (Fig. 4e). Sugars only accumulated in the spike organs during drought. Fructose accumulated in the awn (Fig. 4g), glucose accumulated in all three spike organs (Fig. 4h), and sucrose accumulated in the lemma (Fig. 4i) during drought. For the organic acids, malic acid accumulated in the lemma during drought (Fig. 4j).

## Discussion

The spike (ear) of cereals consists of photosynthetic organs that are important sources of assimilate for grain filling but their response to drought stress is poorly understood. The few previous studies that examined drought response in cereal spikes either focused solely on the awn or on the entire spike as a collective unit [8–10, 50, 53, 54, 57, 58, 68]. Our goal in this study was to characterize the drought response of individual spike organs (awn, lemma, and palea) in barley during the early stage of grain filling and to compare those responses with that of a vegetative organ (i.e., the fifth leaf). We found that these four organs displayed contrasting responses to drought, as indicated by differences in: (1) relative water content (RWC); (2) osmotic potential (Ψ_s_); (3) extent of osmotic adjustment (OA); (4) rates of gas exchange in the awn and fifth leaf; and (5) accumulation of metabolites. Our results suggest that the spike organs are more drought resistant than the fifth leaf, and, among the spike organs, the lemma and palea are more drought resistant than the awn.

### The water status of the fifth leaf and spike organs during drought

The combination of RWC and Ψ_s_ indicates whether plants maintain good hydration during drought through OA. RWC decreased progressively over the four-day drought period in all four organs but the rate of decline in the awn, lemma, and palea was more moderate than that of the fifth leaf (Fig. 1). Similarly, drought reduced Ψ_s_ in all four organs but the difference in Ψ_s_ between control and drought treatments was smallest in the fifth leaf. Further, Ψ_s_ was significantly higher (less negative) in the stressed fifth leaf than the stressed palea (Fig. 2). Consistent with these differences in RWC and Ψ_s_, we found that the lemma, palea, and awn adjusted osmotically to drought and the fifth leaf did not (Table 1, Additional file 1: Table S2). The lack of OA in the fifth leaf suggests that osmolyte accumulation in this organ (Fig. 4) may be due to passive water loss from the cytoplasm during drought. Alternatively, this result may suggest that the 4-day drought treatment caused cellular injury in the fifth leaf. Indeed, osmolyte accumulation is a common symptom of drought-induced cellular damage [69]. Among the spike organs, the awn, lemma, and palea had similar losses in RWC (Fig. 1) and displayed comparable OA (Table 1). The awn had higher (less negative) Ψ_s_ than the lemma and palea during drought; however, this difference was only significant between the awn and palea. Therefore, our results suggest that the spike organs maintain more cellular hydration than the fifth leaf during drought and, to a lesser extent, the lemma and palea maintain more water than the awn.

### The fifth leaf and awn exhibit different gas exchange responses during drought

In addition to their differences in RWC, Ψ_s,_ and OA, the awn and fifth leaf had different rates of gas exchange during the drought treatment. The major difference was the time it took for photosynthesis (*A*) and stomatal conductance (*g*_s_) to decline following the stress. In the fifth leaf, *A* and *g*_s_ sharply decreased on the second day of drought, whereas in the awn, these processes did not show significant decline until the third day of stress (Fig. 3). This suggests that, compared to the awn, the leaf contributes very little assimilate for grain filling during drought. The rapid shut-down of gas exchange in the fifth leaf could be related to its lack of OA (Table 1), which would limit its ability to maintain turgor pressure in the guard cells [70]. Alternatively, drought may have inhibited gas exchange in the fifth leaf at the biochemical level [71]. However, it must be pointed out that gas exchange was not sustained in the awns indefinitely as both organs had comparably low rates of *A* and *g*_s_ on day four of the drought treatment (Fig. 3). The decline in gas exchange in the awn was not because of a lack of OA (Table 1) but rather, was most likely caused by drought-induced inhibition of the photosynthetic metabolism [71]. This interpretation is supported by our previous transcriptome study, which showed down-regulation of photosynthetic genes in the awn of Morex barley on the fourth day of drought [59]. It is worth noting that the high number of awns in the barley spike increases the surface area for photosynthesis [50, 72] and the total assimilate contributed by the awns could still be higher than that of the fifth leaf even on the third or fourth day of drought stress.

We did not measure gas exchange in the lemma or palea because of the challenges associated with accurately measuring this process on these organs. However, our RWC, Ψ_s_, and OA data suggest that these organs are more drought resistant than the awn. Further, we previously showed that the lemma and palea express fewer genes than the awn during drought [59]. Taken together, these evidences suggest that the lemma and palea might maintain higher rates of gas exchange during drought than the fifth leaf or even the awn. Proper measurement of gas exchange in the lemma and palea is needed to test this hypothesis.

### The fifth leaf and spike organs accumulate different metabolites during drought

Suppression of photosynthesis by abiotic stress leads to accumulation of reactive oxygen species (ROS) [73–76]. ROS can destroy nucleic acids, proteins, carbohydrates, and lipids [77]. Drought-induced stomatal closure restricts uptake of CO_2_ and the use of NADPH and ATP in the Calvin cycle, favoring the production of singlet oxygen, superoxide, and H_2_O_2_ in the photosynthetic electron transport chain. Disruption of photosynthesis also increases production of H_2_O_2_ during photorespiration in the peroxisome and the mitochondrial electron transport chain [74, 78, 79]. In addition to their role as osmolytes for turgor maintenance, metabolite accumulation can detoxify ROS and stabilize subcellular structures in drought-stressed tissues.

We detected significant accumulation of ten metabolites in the photosynthetic organs of barley following the 4-day drought treatment (Fig. 4, Additional file 1: Table S2). Metabolite accumulation in the barley cultivar we used (Giza 132) is consistent with other studies [80–87]. Previous studies have shown that the types of osmolytes that accumulate during drought are generally species-specific [81, 84, 86, 88]. Our results expand on this conclusion by showing that osmolyte accumulation during drought is organ-specific in barley (Fig. 4). Accumulation of amino acids during drought is due to active synthesis, inhibition of their degradation, and/or break down of proteins [89–91].

Proline was the only metabolite that accumulated in all four photosynthetic organs during drought (Fig. 4) suggesting that this amino acid plays an important role in the overall drought response of barley. This result is consistent with other studies that detected accumulation of proline in response to drought [83, 92, 93]. Proline serves as an energy source, a stress-related signal [93, 94], and as an osmolyte for turgor maintenance and protection of cellular functions through ROS scavenging and stabilization of subcellular structures [95]. In the cytosol and chloroplasts, proline is synthesized from glutamate by pyrroline-5-carboxylate synthetase (P5CS) and pyrroline-5-carboxylate reductase (P5CR). In the mitochondria, proline is synthesized from arginine catalyzed by arginase and ornithine aminotransferase (OAT) [69]. Proline is degraded to glutamate in the mitochondria by proline dehydrogenase (PDH) and pyrroline-5-carboxylate dehydrogenase (P5CDH) [69]. *P5CS* is up-regulated during drought [96] and *PDH* is down-regulated [97, 98], promoting proline accumulation. *P5CR, arginase,* and *OAT* are up-regulated in the awn, lemma, and palea of barley during drought [59] and these enzymes may be the major players of proline accumulation in the spike.

Five other amino acids (glycine, valine, isoleucine, threonine, and phenylalanine) accumulated in the fifth leaf and variably in the spike organs during drought (Fig. 4). The last step in the biosynthesis of the branched-chain amino acids valine and isoleucine is catalyzed by the enzyme branched-chain aminotransferase (BCAT). This enzyme is also involved in the initial steps of isoleucine catabolism. BCAT maintains the concentration of the branched-chain amino acids below toxic levels by controlling their synthesis and degradation [99]. The *BCAT* gene is inducible by drought [59, 99] and ABA [100]. Threonine (aspartate family) is the substrate for isoleucine biosynthesis. Increased threonine concentration in the fifth leaf and awn (Fig. 4) might also have contributed to the accumulation of isoleucine during drought.

The aromatic amino acid phenylalanine accumulated in the fifth leaf and lemma (Fig. 4). Aromatic amino acids are synthesized via the shikimate pathway and serve as precursors for several secondary metabolites. The accumulation of phenylalanine in the lemma is inconsistent with our previous transcriptome analysis, which showed no change in expression of aromatic amino acid biosynthesis genes in the lemma and down-regulation in the awn during drought [59]. Nevertheless, phenylalanine accumulation in the fifth leaf is consistent with reports in other species, such as maize, during drought [101].

Sugars are important sources of carbon and energy [102]. They also serve as signal molecules [2, 103–105] and osmolytes [102, 106, 107]. We detected accumulation of glucose in all three organs of the spike, suggesting it plays an important role in the overall drought response of the spike. Fructose accumulated only in the awn and sucrose accumulated only in the lemma during drought (Fig. 4). Accumulation of different sugars may suggest that each spike organ uses different osmolytes for drought resistance. Nevertheless, accumulation of sugars in the barley variety we used is consistent with accumulation in other species during drought [85, 108, 109].

The organic acid malate is an intermediate in the citric acid (tricarboxylic acid) cycle, the glyoxylate cycle, and photosynthesis (C_4_ and Crassulacean acid metabolism, CAM). Malate plays a central role in plant metabolism and homeostasis, including providing a carbon skeleton for amino acid biosynthesis, as an osmolyte, regulation of pH homeostasis, as a root exudate during phosphorus deficiency, and as a reducing equivalent shuttled between subcellular compartments [110–113]. In our study, malate accumulated only in the stressed lemma (Fig. 4) and this agrees with accumulation in maize [114] and wheat [85] during drought. Accumulation of malate is consistent with up-regulation of *MDH* (malate dehydrogenase) in the lemma during drought [59, 115]. MDH catalyzes the interconversion of malate and oxaloacetate and accumulation of malate may suggest that MDH predominantly catalyzes the conversion of oxaloacetate to malate in the lemma during drought.

## Conclusions

In this study, we showed that the spike organs (lemma, palea, and awn) and vegetative organs (fifth leaf) of barley respond differently to drought at the grain filling stage. Based on differences in RWC, Ψ_s_, extent of OA, gas exchange, and metabolite accumulation, we conclude that the spike organs of barley maintain more cellular hydration than the fifth leaf, and, to a lesser extent, the lemma and palea retain more water than the awn during drought. We propose that the spike organs employ two strategies for coping with drought: drought avoidance via osmotic adjustment and drought tolerance through ROS scavenging and stabilization of macromolecules.

## Additional file

Additional file 1: Table S1. ANOVA results for the effects of treatment, organ, time, and their interactions on RWC and gas exchange. **Table S2.** ANOVA results for the effect of treatment, organ, and their interaction on ten metabolites. (DOCX 18 kb)

## Abbreviations

*A*: Photosynthesis
*g*_s_: Stomatal conductance
MPa: Megapascal
OA: Osmotic adjustment
RWC: Relative water content
Ψ_s_: Osmotic potential
