# Supplementary material for: The fifth leaf and spike organs of barley (Hordeum vulgare L.) display different physiological and metabolic responses to drought stress
Source: BMC Plant Biol. 2016 Nov 9;16:248. doi: 10.1186/s12870-016-0922-1 (PMC5103489; doi:10.1186/s12870-016-0922-1)
Supplement: Additional file 1: Table S1. — ANOVA results for the effects of treatment, organ, time, and their interactions on RWC and gas exchange. Table S2. ANOVA results for the effect of treatment, organ, and their interaction on ten metabolites. (DOCX 18 kb) [file 12870_2016_922_MOESM1_ESM.docx]

**Additional files**

**Additional file 1: Table S1**. ANOVA results for the effects of treatment, organ, time, and their interactions on RWC and gas exchange.

| **Parameter measured** | **Effect** | **Num df** | **Den df** | **F** | **P** |
| --- | --- | --- | --- | --- | --- |
| RWC | Treatment | 1 | 4 | 61.85 | 0.0014 |
|  | Organ | 3 | 60 | 63.70 | <0.0001 |
|  | Treatment × organ | 3 | 60 | 29.04 | <0.0001 |
|  | Time | 3 | 60 | 76.46 | <0.0001 |
|  | Time × treatment | 3 | 60 | 98.10 | <0.0001 |
|  | Time × organ | 9 | 60 | 6.95 | <0.0001 |
|  | Time × treatment × organ | 9 | 60 | 4.32 | 0.0002 |
| *Ψ_s_* | Organ | 3 | 31 | 7.77 | 0.0005 |
|  | Treatment | 1 | 31 | 1101.65 | <0.0001 |
|  | Organ × treatment | 3 | 31 | 12.79 | <0.0001 |
| *A* | Treatment | 1 | 4 | 116.31 | 0.0004 |
|  | Organ | 1 | 28 | 22.59 | <0.0001 |
|  | Treatment × organ | 1 | 28 | 3.42 | 0.0748 |
|  | Time | 3 | 28 | 11.12 | <0.0001 |
|  | Time × treatment | 3 | 28 | 11.51 | <0.0001 |
|  | Time × organ | 3 | 28 | 8.21 | 0.0004 |
|  | Time × treatment × organ | 3 | 28 | 1.70 | 0.1888 |
| *g*_s_ | Treatment | 1 | 4 | 72.64 | 0.0010 |
|  | Organ | 1 | 28 | 27.93 | <0.0001 |
|  | Treatment × organ | 1 | 28 | 16.82 | <0.0003 |
|  | Time | 3 | 28 | 3.68 | 0.0238 |
|  | Time × treatment | 3 | 28 | 28.71 | <0.0001 |
|  | Time × organ | 3 | 28 | 3.62 | 0.0251 |
|  | Time × treatment × organ | 3 | 28 | 3.53 | 0.0276 |

ANOVA was performed to determine the effects of treatment (control vs. drought), organ, time (day of treatment), and their interactions on RWC, *A*, and *g*_s_ and the effects of treatment, organ, and their interaction on Ψ_s_. RWC, *A*, and *g*_s_ were measured each day of the four day treatment from plants grown in a completely randomized design. Time was the repeated measures factor. Ψ_s_ was measured on each of the four organs on the fourth day of treatment from plants grown in a randomized complete block design. Values are: numerator degrees of freedom (Num df), denominator degrees of freedom (Den df), F-statistic (F), and p-value (P).

**Additional file 1: Table S2**. ANOVA results for the effect of treatment, organ, and their interaction on ten metabolites.

| **Metabolite** | **Effect** | **Num df** | **Den df** | **F** | **P** |
| --- | --- | --- | --- | --- | --- |
| Glycine | Organ | 3 | 34 | 5.34 | 0.0040 |
|  | Treatment | 1 | 34 | 65.69 | <0.0001 |
|  | Organ × treatment | 3 | 34 | 5.77 | 0.0027 |
| Valine | Organ | 3 | 34 | 11.97 | <0.0001 |
|  | Treatment | 1 | 34 | 53.26 | <0.0001 |
|  | Organ × treatment | 3 | 34 | 0.87 | 0.4664 |
| Isoleucine | Organ | 3 | 34 | 13.32 | <0.0001 |
|  | Treatment | 1 | 34 | 33.09 | <0.0001 |
|  | Organ × treatment | 3 | 34 | 2.90 | 0.0491 |
| Threonine | Organ | 3 | 34 | 11.75 | <0.0001 |
|  | Treatment | 1 | 34 | 39.71 | <0.0001 |
|  | Organ × treatment | 3 | 34 | 2.94 | 0.0468 |
| Phenylalanine | Organ | 3 | 34 | 2.56 | 0.0711 |
|  | Treatment | 1 | 34 | 50.41 | <0.0001 |
|  | Organ × treatment | 3 | 34 | 2.08 | 0.1208 |
| Proline | Organ | 3 | 34 | 11.30 | <0.0001 |
|  | Treatment | 1 | 34 | 337.52 | <0.0001 |
|  | Organ × treatment | 3 | 34 | 6.58 | 0.0013 |
| Fructose | Organ | 3 | 34 | 23.48 | <0.0001 |
|  | Treatment | 1 | 34 | 24.49 | <0.0001 |
|  | Organ × treatment | 3 | 34 | 2.87 | 0.0508 |
| Glucose | Organ | 3 | 34 | 45.61 | <0.0001 |
|  | Treatment | 1 | 34 | 110.25 | <0.0001 |
|  | Organ × treatment | 3 | 34 | 4.40 | 0.0101 |
| Sucrose | Organ | 3 | 34 | 6.86 | 0.0010 |
|  | Treatment | 1 | 34 | 5.82 | 0.0214 |
|  | Organ × treatment | 3 | 34 | 5.90 | 0.0024 |
| Malic acid | Organ | 3 | 34 | 13.38 | <0.0001 |
|  | Treatment | 1 | 34 | 23.66 | <0.0001 |
|  | Organ × treatment | 3 | 34 | 0.93 | 0.4366 |

Metabolite content was measured for the fifth leaf, awn, lemma, and palea on the fourth day of drought treatment from plants grown in a randomized complete block design. Values are: numerator degrees of freedom (Num df), denominator degrees of freedom (Den df), F-statistic (F), and p-value (P).
